# Teaching Without Harm: The Ethics of Performing Posthumous Procedures on the Newly Deceased

**DOI:** 10.7759/cureus.11855

**Published:** 2020-12-02

**Authors:** Aarabhi S Rajagopal, Thomas H Champney

**Affiliations:** 1 Pediatrics, Ann & Robert H. Lurie Children's Hospital of Chicago, Chicago, USA; 2 Institute for Bioethics and Health Policy, University of Miami Miller School of Medicine, Miami, USA; 3 Cell Biology, University of Miami Miller School of Medicine, Miami, USA

**Keywords:** ethics, medical education, teaching, death, nearly dead, cadaver, attitude to death, informed consent, donation

## Abstract

Physicians must be proficient in and efficient at various lifesaving and life-sustaining procedures. Multiple methods exist to teach these skills to inexperienced medical professionals, ranging from lectures to practical models to live patients. Proficiency and prior knowledge are especially important when novice medical trainees first perform these procedures because of the increased risk of harm in these high-stakes scenarios. To mitigate inherent risks, many medical centers controversially advocate and allow the use of newly deceased patients to practice, teach, and perfect these procedures. As a result, this type of experience facilitates medical training and competency while simultaneously avoiding physical harm to living patients. Nonetheless, it raises numerous ethical and legal considerations, including concerns of damage to the doctor-patient relationship.

This manuscript aims to comprehensively review the ethicality of practicing postmortem procedures and its current debate regarding the role and type of consent. This is followed by examining scenarios where the prior patient or postmortem surrogate consent is required for procedures that do not benefit the patient, including organ donation, cadaver donation, and brain tissue donation. Using these scenarios as a framework, best practices for gaining permission to use the newly deceased for medical training purposes are offered.

Procedures on deceased patients should always be done under competent supervision in a structured manner, with comprehensive explanations to encourage accountability and professionalism and prevent misuse. Informed consent for all educational procedures must be obtained by individuals separate from the treatment team. However, exceptions to this standard could be made in pediatrics (especially in the neonatal intensive care unit) given the intimate relationship between providers and parents. Depending on the situation, consent should be obtained from the patient and/or their family, with separate documentation provided to create awareness. All relative parties should be consented after receiving appropriate time to process to prevent further emotional compromise. If there are concerns about jeopardizing the family and creating further burdens, they should not be approached.

## Introduction and background

The medical profession is an amalgamation of art, humanism, and science one must master to become a skilled clinician. The scientific aspects of medicine constantly grow, with new discoveries, diverse research, and expanding literature being produced at an ever-growing pace [1]. Simultaneously, the art and humanism of medicine allow physicians to foster meaningful relationships with their patients while diagnosing illnesses, providing treatments, and performing procedures as needed. Yet, physicians are bound by the Hippocratic oath and the nature of their job to not only treat individual patients but also advance the health of society. Thus, the obligations of physicians include treating maladies, relieving suffering and pain, and preventing any harm from occurring [1, 2].

The doctrine of “do no harm” is especially relevant to physicians’ procedural skills since many minimally invasive procedures help to sustain and save lives. Doctors are expected to obtain these skills as quickly as possible while achieving success with minimal mistakes throughout the process. The need for proficiency and efficiency has led to the development of multiple models to teach medical trainees, including virtual reality programs, animal models, cadaver donors, and live patients. Many medical centers also allow trainees to practice procedures on newly deceased patients. For novice trainees, this practice allows them to attain procedural skills with an understanding of varying circumstances and anatomic variants while causing minimal harm to deceased patients [1].

Despite this method serving a dual purpose by teaching physicians and allowing them to serve the public, there are a myriad of ethical and legal concerns to be considered. Previous studies have examined the nature of this practice, deeming it ethical as long as valid consent is obtained. However, in practice, consent is rarely obtained because of the ramifications and logistics involved. Past clinical and ethical discussions have also shed little light on the ideal method of consent. This juxtaposition prompted a holistic review regarding this practice, its ethical considerations, and the role of prior patient or postmortem surrogate consent in comparison to similar practices that do not benefit the patient. Examining these facets will subsequently assist in delineating the best ethical approach overall.

## Review

Ethical considerations to using newly deceased patients

In the United States, practicing procedures that do not benefit the patient for the sole purpose of gaining proficiency occurs in many training programs. Almost two-thirds of emergency training programs employ this practice to teach their trainees, along with an unreported number of medical, obstetric and gynecologic, surgical, and pediatric training programs [2]. Typically, these trainees are residents and fellows. Medical students are often not given the opportunity due to their limited training and the higher risks of harm [2, 3]. All of the procedures being mastered are life-saving interventions meant to benefit patients when they are critically ill, but they are also interventions that have a high risk of harm if not done properly. Most commonly, this includes endotracheal intubation, cricothyrotomy, laryngeal mask airway placement, central and peripheral arterial and venous line placement, needle decompression, pericardiocentesis, and cervical traction [1].

In most cases, procedures occur in the window period between “time of death” and subsequent notification to the appropriate parties. Typically, these procedures are done on adult patients who die in the emergency department or the intensive care unit, most commonly of cardiac death [3]. In certain scenarios, they are also done on neonates who die in the neonatal intensive care unit [4, 5]. The selected patients are often those who are not eligible for organ donation, given the time restrictions and specifications related to organ procurement. Similarly, patients who are in a persistent vegetative state do not qualify because they are not deceased, and they are often in long-term care facilities when they die. Patients who are “nearly dead” (i.e., those undergoing resuscitation) are also not considered to be ideal candidates because they are the ones who need the procedures to save their lives [1].

The goal of using newly deceased patients to practice and perform life-saving interventions is multifactorial, with the primary aim of helping trainees become proficient at stabilizing patients in critical situations. To gain this proficiency, one must have not only the knowledge of how to perform the procedure and its indications, benefits, and risks but also the experience of performing it in varying circumstances. There are numerous dynamic variables with each critical situation, including differing stress levels, different patient conditions (i.e., the spectrum of stable versus unstable), and anatomical variations. Many life-saving interventions are also often performed in the “pre-hospital” setting by emergency medical services, reducing opportunities for physicians in training to gain procedural skills. At the same time, many of these skills cannot be learned partially or “on the fly” given their complexity, and not all clinicians have dedicated, thorough training to help increase their efficiency and success rates. Given the inherent risk of practicing on live patients, this alternative method using newly deceased patients allows trainees to learn procedural skills while minimizing any potential harm that may occur. In this manner, physicians-in-training can advance the important common social goal of medical competency and contribute to the social atmosphere dedicated towards reducing medical errors [1, 6, 7].

In recent times, this practice has decreased in frequency due to bioethicists' and clinicians' efforts to develop ethical standards across the medical community, advocating for patient autonomy and respect [2, 8]. However, training programs that still partake in it face a unique quandary between the opportunity to gain competency in their procedural skills and their duty and respect towards the recently deceased patient and their families. Because of its logic and necessity, many in the medical community continue to advocate for trainees to practice their skills via posthumous procedures [2, 3]. In particular, this practice allows trainees to learn procedures and fulfill their obligations to the public without causing significant harm and risk to living patients. This is especially important for family physicians in rural settings with limited resources, given the significantly large population coverage and sparsity of hospitals [1, 2]. In line with this, Joel Feinberg argues that the body should not be protected “at the expense of the vital human interests of a real person” [9]. This reasoning is why many neonatal critical care centers (NICUs) continue to advocate for pediatric trainees to engage in this practice to sharpen their skills in endotracheal intubation or umbilical line placement, as these procedures are lifesaving interventions [4]. At the same time, Feinberg argues that denying physicians the skills to prevent more patients from dying ultimately creates a poor sign of respect to a sacred symbol [9]. Many additionally argue that respect for others is still present throughout this process, as procedures are conducted while showing deference to the deceased and their bodies [1-3]. Thus, clinicians who practice procedures on the recently deceased will ultimately pay homage to the deceased by not only gaining skills to save more patients in the future but also through the remembrance of those who sacrificed themselves for their education.

While there are many alternatives trainees may use to perfect their procedural skills, the associated drawbacks lead many to support practicing on the newly deceased. In many scenarios, donated cadavers have been considered the ideal alternative, given the ability to practice on human tissues from those who willingly chose to donate their bodies for science. However, stiff tissues secondary to embalming and rapid cooling prevent appropriate visualization and determination of certain structures. This subsequently prevents appropriate positioning of certain lifesaving devices, such as endotracheal tubes or central lines. Likewise, animal models cannot approximate the actual difficulty of many procedures due to the stiff tissues and minimal representation of human anatomy. Nevertheless, new embalming techniques (e.g., Thiel embalming) have made it easier to teach procedures, as they allow tissues to remain more life-like. Many medical centers have also developed robust simulation centers with non-living, programmable models. These computer-simulated mannequins can often give the experience of a procedure because of their ability to simulate realistic emergency situations. Yet, the limited representation of the human form, secondary to the high costs of virtual reality models and our current technological capabilities, prevent simulated patients from being perfect examples [1, 3].

One other alternative some have considered is practicing on live patients to maintain the authenticity of the tissues and expose trainees to varying real situations. One such group of living patients are those placed under anesthesia in the operating room. Another such group consists of patients who need the procedure in question, where trainees practice and learn under direct supervision multiple times, subsequently honing their skills. The last group of living patients is those who are “in limbo,” where they cannot be resuscitated fully, but death has not been pronounced. In these scenarios, the resuscitation process is prolonged until all trainees have a chance to practice cardiopulmonary resuscitation (CPR) to restart the heart or restore the blood pressure to normal levels [10]. Yet, practicing on living patients poses a great risk and an even greater potential of harm. In general, patients and/or their families will additionally have to pay for the costs of all procedures done and equipment used via medical bills and insurance claims, creating a significant financial burden on top of any emotional turmoil. In the case of patients placed under anesthesia, they derive little to no benefit from receiving these procedures, although they legally consented to the medical trainees’ participation to benefit their care. Thus, performing on these patients has largely been deemed unethical, given the increased risk of harm for an unnecessary procedure [1, 2, 11]. However, the question of harm is especially true for patients in limbo, as procedures are repeatedly performed on a still-living patient who does not need them. Repeating these unnecessary procedures not only prolongs the process of dying but also leads to severe systemic damage internally, creating significant harm. In turn, the profound disregard for living patients’ rights and autonomy can fundamentally alter trainees’ perspectives regarding patient care, shifting away from the “patient first” philosophy [1, 3, 6].

Several argue against trainees performing posthumous procedures to practice and gain skills because of the inherent lack of respect towards the rights of the decedent and their survivors. This disregard can further erode a physician’s sense of empathy, damaging the doctor-patient relationship because a detached attitude towards human life develops. This practice also leads to the degradation of trainees’ moral and ethical views of patients as they repeatedly and rapidly shift from saving others’ lives to using them as “practice mannequins” [11]. Furthermore, manipulating the body in the immediate aftermath may subsequently impede the medical examiner’s efforts at uncovering the cause of death, as they control the initial body disposition when the death is sudden, unexpected, or violent in a manner [1]. Many also argue that this practice goes against physicians’ ethical obligations towards their communities, as some cultures strictly disallow manipulation of the dead. Even though religious and cultural traditions are on a spectrum, desecrating the deceased through posthumous manipulation would violate several core beliefs. For example, several Native American and Asian cultures view the body as sacred in all its forms and believe that it must remain intact after death for spiritual reincarnation to occur. Similarly, Orthodox Judaism prohibits the mutilation of the corpse, as well as deriving benefit from the deceased [11]. From a scientific standpoint, many argue against trainees practicing via posthumous procedures because empirical studies have never significantly indicated that their skill level increases by being able to practice in this manner [2]. Limiting this practice will also stimulate research on better simulated models, allowing higher functioning alternatives to be developed that prevent deceased and living patients from experiencing significant harm [1, 2].

Ethical considerations regarding the role of consent

Among the considerations for respect for others, honesty, and societal obligation, previous studies have also debated the role of consent within this practice to increase its ethical integrity [1, 2, 4, 6, 10-12]. Many ethicists have posed that trainees practicing procedures via the newly deceased are ethical, as long as consent is involved [6, 10, 12]. Many professional organizations also formally recommend asking for valid consent before trainees can practice procedures, including the Society of Academic Emergency Medicine, the American Heart Association, and the American Medical Association (AMA) [2, 3]. Similarly, when polling the public regarding this practice, Manifold found that 75% of families would consent to procedures, with 60% percent becoming upset if procedures were done without consent [11]. Oman and colleagues found similar results, with 54% believing that teaching procedures in this manner were acceptable and 80% believing that consent was necessary [13]. Despite this consensus, McNamara and others found that of the 63% of emergency medicine training programs and 58% of neonatal critical care centers that allow procedures to be practiced on patients after death, only 10% obtained family consent [14].

Given the dichotomy between ethical consensus and clinical practice, there continues to be a debate on whether consent is truly required for this practice. From a physician's perspective, having consent allows procedures to be taught outside of the patient care setting. In turn, this creates the opportunity for structured teaching, allowing trainees to truly learn the procedure(s) before they put themselves or a patient at risk. Structured teaching sessions also ensure that the trainee will not only learn the technical steps involved, but also the indications, benefits, and risks for several procedures [1]. From an ethical perspective, many argue consent is required because practicing these procedures without consent violates the principles of non-maleficence, integrity, and honesty. Under an “aegis” of justice, physicians should obtain consent in accordance with their professional code of ethics. Moreover, having consent would allow this practice to meet the criteria of universalizability posed by Kant, rendering it morally acceptable since it would be open to debate and able to be performed by anyone [2, 15]. Others argue that consent is required because it allows physicians to respect the rights of the decedent while preventing the trust placed in physicians from being undermined [1]. Many additionally advocate for consent because the “don’t ask, don’t tell” status quo has additional consequences beyond violating physicians’ ethical obligation towards accountability. Consent also helps to remove any further emotional trauma survivors may experience by providing assurances and comfort about the treatment of the deceased [16, 17].

Consenting also helps practitioners appropriately disclose and thoroughly explain the process to families while bridging any gaps related to specific religious or cultural beliefs and views about the patient’s medical care [2, 3]. In particular, Morag and colleagues found that religion and ethnicity played a large role in whether someone would consent or not, where respondents in Brooklyn (non-white, Catholic) were less willing to grant permission than those in Oslo (white, non-religious). They also found that respondents in Brooklyn tended to respond more negatively compared to those in Oslo when asked for consent, suggesting that varying cultural attitudes regarding death played a significant role as well. Across both populations, consent was more likely to be obtained for procedures done postmortem on adult patients in comparison to pediatric patients. However, there was no difference in the agreement rates between both populations based on age, sex, or education level [4]. Similarly, Mirzazadeh and coworkers found that Iranian patients and their relatives were less likely to agree to endotracheal intubation being performed posthumously. They accounted for the difference in rates between Iran and Western countries due to religious and cultural differences, relative health literacy, age, and sex [18]. In a more recent small study of Iranian medical students and residents, the ethical, professional, and legal concerns around the use of the recently deceased were examined, with approximately half of the respondents expressing concern over procedures being done [8].

At the same time, many posit that consent is not ethically required because the tenets of non-maleficence and autonomy do not survive death [6]. As autonomy and informed consent are founded upon the basis of individualism, they cannot be enacted upon the recently deceased because corpses are not individuals. However, autonomy can be preserved posthumously through the application of advanced directives or living consent obtained prior to death. If autonomy were to be extended via informed consent for the recently deceased, physicians would subsequently be creating “ethical fiction,” expanding the principle beyond its meaning. Similarly, the concept that harm can occur to the dead via this practice has been characterized as “legal fiction,” as there is no inherent risk to recently deceased patients if they underwent these procedures [1]. Legally, many states do not require survivors’ consent to harvest and use organs, such as corneas and pituitary glands, due to the principle of presumed consent. Additionally, while there are various statutes and laws regarding corpse mistreatment in several states, none prohibit the practice of posthumous procedures for teaching purposes, and there have not been any legal cases that are directly related to this practice [16]. On the other hand, many believe that standard informed consent is not adequate, as it is typically done to educate and discuss treatment options. Beyond this, patients who undergo an invasive procedure are rarely given true, adequate informed consent. Furthermore, patients who enter teaching hospitals implicitly agree to participate in resident training in all forms, giving permission for trainees to practice on their bodies regardless of whether they are alive or fully aware of their consent. In conjunction with this, many argue that enacting formal informed consent is not necessary for physicians because the practice has been occurring under presumed consent for many years. Physicians are also afraid to ask for permission in this context due to the further burden placed on the family during a time of mourning. Thus, having physicians formally request permission may lead to one of two consequences: a) they will be less likely to ask (and thus less likely to gain practice) or b) they practice without asking, leading to further ethical quandary. In this manner, consent potentially acts as a barrier to maintaining these skills, ultimately leading to a societal disservice because of the subsequent limitation of the practice [1, 3].

In conjunction with the debate regarding the role of consent, multiple methods of consent have been suggested, but a consensus has yet to be reached. In particular, the AMA Council on Ethical and Judicial Affairs recommends that doctors should only perform procedures if they can determine a patient’s preferences prior to death or obtain postmortem surrogate consent from the family [19]. Previous studies have also suggested that consent should be obtained from relatives because families often respond positively to such requests since they are given the opportunity to be altruistic [11, 12, 14]. For example, Manifold et al. found that 75% of polled families would consent to various procedures being done, while McNamara and others found that 59% of families in the NICU consented to posthumous retrograde tracheal intubation [11, 14]. Having families consent for this practice allows them to have something positive come from the loss of a loved one while doing something that benefits others. Additionally, survivors have autonomy after a patient’s death because they are the de facto decision-makers regarding the care of the body. By performing invasive procedures without their consent, even if it is for training purposes, it violates their right to make decisions with regards to their loved one’s body [4, 18].

On the other hand, some argue that consent should come from the patients. In particular, Mirzazadeh and colleagues found that 76% of patients and 91% of companions believed it was necessary to acquire patient consent for postmortem procedures [18]. Furthermore, consenting the patients allows them to express their wishes and have them abided by while preventing survivors from undergoing further emotional distress or being burdened with additional decisions [2, 20]. Studies have also shown that the approval rate for performing procedures on one’s own body is high, likely due to a sense of ownership [1]. For instance, Alden, et al. found that 74% of respondents were willing to undergo intubation, while 67% were willing to undergo chest tube insertion [21]. Morag and colleagues also found that respondents in Oslo were more likely to permit procedures being practiced on themselves rather than on a relative [4]. Yet, other studies suggest that both patients and their families should be consented for this practice. Mirzazadeh and others found that 70% of patients and 71% of companions believed that obtaining relatives’ consent was necessary, even when prior patient consent had been obtained. Almost half of the patients additionally agreed to endotracheal intubation being practiced on a relative’s body, knowing that the deceased had consented prior to death. By consenting in this manner, decision-making became easier since everyone’s viewpoint is considered, thus increasing the chances of approval for the procedure [18].

In addition to the debate on whom should be consented, there has also been debate on who should enact the consent process, as well as when, how, and which situations to consent. Several studies have shown that families tend to respond positively when they are asked by physicians separate from the medical care team, especially by those who are trained to ask in such situations [2]. However, in certain cases, families respond more positively to physicians that they know. For example, Benefield and colleagues found that 73% of families in the NICU consented for trainees to practice posthumous neonatal intubation when approached by a physician that had been part of the medical care team [5]. In terms of timing, many have suggested that families be consented in the perimortem or postmortem period, as that is a time-point where they are able to make a voluntary, informed decision [2, 16, 20]. For patient consent, previous studies suggest that it should be done antemortem, but few have suggested at which time-point during clinical practice (e.g., upon presentation, prior to the situation becoming critical, etc.) [2, 4]. For those who advocate for consenting both patients and their families, Mirzazadeh and colleagues found that participants believed consent should be obtained from both parties for all practice procedures [18]. In contrast, Morag and coworkers found that participants believed that patient consent is sufficient for non-invasive procedures, while invasive procedures should have consent from patients and their families [4].

Moreover, multiple methods of consent have been discussed, with the majority suggesting either verbal informed consent, formally documented informed consent, or a consent process like that of organ donation. Much of the public also supports a consent process similar to the advanced directives for organ donation, as it allows an official declaration of consent a priori. Enacting this consent process would allow the pool of donors for procedural practice to increase. Having a separate consent process from clinical practice also avoids several potential conflicts. This includes regional and demographic differences between patients, a need for family consent, the involvement of hospital policies, and the practice of prolonging death on live patients via a slowed resuscitation process. However, some suggest that formal a priori consent would have little effect on the agreement rate for this practice based on known experiences with organ donation [2, 4].

Comparable processes: organ donation

In contending with this complex issue, one possible framework for developing a standard informed consent process for the practice of using newly deceased patients for posthumous procedures can be found by examining the deceased organ donation process. As of March 2020, more than 112,000 patients in the United States (US) require organ transplantation, with 20 patients dying every day waiting for their transplants [22]. As the demand for organ transplantation has grown, so has the need for organ donation. With organ donation, the main goal is to recruit healthy individuals to increase the number of organs available for transplantation, thus reducing the gap between supply and demand for organs across the world [22, 23]. In America, deceased organ donation involves an “opt-in” process, where interested adults register with their state’s department of motor vehicle services to become an organ donor. During the registration process, donors undergo informed consent, learning about the process of organ donation, its risks and benefits, and the overall impact. Once registered, donors’ statuses become listed on their driver’s licenses, and they may choose to unregister at any time. In some states, persons younger than 18 can also register, undergoing an analogous process. When a donor dies (either by cardiac or cerebral means), the medical team in charge initially places them on artificial or mechanical support to ensure continued perfusion to maintain organ integrity. Thereafter, they contact the local organ procurement organization (OPO), providing information about the donor and their circumstances. As a unique, separate entity, the OPO ensures that the deceased patient is a registered donor and evaluates them to confirm that their organs are healthy enough for procurement. They then counsel families regarding the organ recovery process, as well as explaining the future impact of their loved one. This process serves as legal consent, in addition to the informed consent donors go through a priori. In cases where the deceased patients eligible for organ procurement are unregistered adult donors or younger than 18, OPOs obtain informed consent from the next of kin. In this case, the next of kin must consent before organs are retrieved, even in the face of documentation that the deceased wanted to be a donor. Once consent is obtained, the organs to be donated are recovered, transferred to the hospital of the organ recipient, and transplanted [23, 24].

In many other countries, deceased organ donation occurs via an “opt-out” process, invoking the concept of presumed consent. In this case, every eligible person is automatically registered as an organ donor, and organ donation is legally considered the default option at the time of the death. Those who do not wish to donate their organs must explicitly opt-out of the process, signing an informed consent of refusal. In these countries, donor rates are as high as 90% (compared to 15-20% in the United States), with many viewing organ donation as a meaningful act that fulfills one’s duty to society. Given the notable discrepancy between donor rates in an “opt-in” vs. “opt-out” process, many have advocated for switching the US’ donor consent process to lead more people towards organ donation. Others have suggested adults make their wishes known in formal, mandated documentation that must be honored upon death, regardless of whether they are registered or not. However, some have argued against this because it denies the families’ rights to have input regarding decisions made about the use of their loved one’s organs [25].

Comparable processes: anatomical donation

In addition to the deceased organ donation process, the cadaveric anatomical donation process provides a framework to develop a standard informed consent process for the practice of using newly deceased patients for posthumous procedures. With cadaver donation, donors’ bodies are used for educational or research purposes to advance medical science and health initiatives, including medical education and training, forensic sciences, vehicle safety, and the development of protective equipment. Often, organizations will place restrictions on who may donate and the condition of accepted bodies to ensure that ideal donors are selected. Typically, this consists of donors who are within an ideal weight range, negative for communicable diseases, and have not undergone an autopsy, trauma, recent surgeries, and/or organ removal [26, 27].

With the anatomical donation, adult donors register with an anatomical gift program or organization of their choice, undergoing informed consent regarding the process, requirements, and risks and benefits of whole-body donation. The consent process also includes an authorization for disposition after donors have served their purpose. After registering, donors receive either a registration card or a declaration form that describes their intent to donate. Pediatric cadaveric donation to a program involves an analogous process, where familial authorization and consent is required prior to accepting whole body donation of children and fetuses. Interested persons are strongly encouraged to discuss their wishes with their family and doctors, ensuring that their wishes are appropriately carried out at the time of death. Programs must also be informed of any changes to a donor’s health after the time of consent, given the restrictions placed on cadaveric donation [28, 29]. At the time of death, a third party or the next of kin must inform the organization about the donor’s death and provide contact information for the medical professionals involved in the donor’s care to certify that they meet the medical criteria for donation. Family members or a designated healthcare proxy then need to submit a signed and witnessed consent form, as well as a copy of the death certificate. Once secondary consent is obtained, the donor’s body is donated to the organization and subsequently studied. Following the study of their bodies, donors are typically cremated, with the ashes returned to families upon their request. Like organ donation, living donors can withdraw from the program at any time. In certain cases, families may have the right to revoke intent of donation, even if the potential donor was previously registered, depending on the policies of the anatomical gift program [27, 29].

Comparable processes: brain tissue donation

A newly burgeoning field, brain tissue donation for medical discoveries provides another framework, where the provision of this service similarly does not directly affect or benefit the patient. With brain tissue donation, tissues are given for research purposes to not only better understand normal brain matter but also the nature of a variety of neurological and psychiatric disorders. Like cadaveric donation, organizations involved with brain tissue donation place restrictions on acceptable diagnoses to ensure ideal tissues are received. Examples of acceptable diagnoses fall into broad categories, including psychiatric, neurocognitive, neurodevelopmental, and neurogenetic disorders [30].

The consent process for brain tissue donation is analogous to that of cadaveric anatomical donation, where interested adults register with an organization or program of their choice. Parents or legal guardians may register their children as potential donors. During the registration process, they undergo informed consent with explanations of the donation process and requirements, benefits and risks, and the postmortem process. After registering, donors will either receive a registration card or a form stating their intent to donate. Donors are highly encouraged to speak with their families regarding their wishes, ensuring that their desires are appropriately pursued posthumously. At the time of death, family members or funeral home must contact the program to inform them about the donor’s death and subsequently consent to the donation in an informed manner, secondarily authorizing the donation. They also provide consent for releasing the donor’s medical records to investigators, ensuring that appropriate studies can take place. Once the consent process has been completed, pathologists organize the recovery and transport of the brain tissue, and investigators subsequently preserve and study the specimens [31].

Best practices for medical trainees performing post-humous procedures

In general, procedures should always be done with the utmost respect under the guidance of those with significant experience performing them. It is also important that they are taught in a structured manner with explanations of their indications, utility, benefits, and risks. Trainees should avoid practicing these invasive procedures on the newly deceased without supervision when opportunities present in an inpatient setting, as that will ultimately lead to more harm. Enacting competent supervision allows procedures to be taught appropriately and efficiently while also creating an environment of professionalism. Having accountability in this manner also prevents harm and misuse from occurring to the deceased patient’s body, ensuring that all proceedings occur with the utmost respect and dignity. Following all procedures, the bodies should be released to the family or disposed of properly with the offer to return the ashes to their loved ones. To safeguard against the ethical qualms, this practice poses, consent for all planned educational procedures must be obtained. Following the principles of valid informed consent, consent should be voluntary, comprehensive, and culturally sensitive, explaining what will be done, its utility, and the risks and benefits for each procedure that is performed. Ideally, consent should be performed early in the process, such as the time of admission to the hospital, to allow an extensive discussion without emotional compromise. If not, then it should be done at a time when patients and/or their family members seem approachable after grieving or “coming to terms” appropriately with a critical change in health. If they are not approachable, then consent should not occur at that time because the long-term emotional health of a family supersedes any teaching opportunity and its potential benefits. Preferably, written consent should explicitly be given to the necessary parties as separate documentation, but the sensitivity of the situation may predominantly advocate for verbal informed consent. Regardless of the manner of consent, physicians should document the discussion briefly. If consent cannot be obtained, then the planned educational procedures should not be performed. In this manner, the standard for living patients will be equivocally applied to dying patients, preserving the tenets of respect even with the reduced risk of harm. Having a formal consent process will allow all concerns to be addressed while obtaining a proper understanding of the practice as a whole. Without consent, there is a significant risk of damage to the doctor-patient relationship since it can erode trainees’ empathy and allow a disrespectful attitude towards human life to develop.

Furthermore, those who obtain consent should be separate from the primary medical team to ensure that impartiality exists and to prevent any conflicts of interest that may occur. An exception can be made for situations involving pediatric patients, as the close physician-parent-patient relationship allows for better responses to the consent process. In turn, the consenting parties agreeing to this practice should differ depending on the extent of the procedures being performed. For minimally invasive procedures, the patient should give consent, while patients and their family members (e.g., spouse or children) should also consent for more invasive procedures. In any situation where the patient cannot be consented, surrogate consent should be obtained from the next of kin. For all pediatric patients, consent should be obtained from parents or legal guardians to ensure that ethical and legal principles are followed. Regardless, all consenting parties should receive or have documentation to provide awareness of their decision to family members and physicians involved in the care of the patient, especially for situations where only patient consent is obtained.

Given its high prevalence, it is imperative that knowledge of the practice of using newly deceased patients for posthumous procedures becomes more public within the medical community. By doing so, we ensure that training programs develop policies and guidelines that clearly delineate the extent of the practice, preventing it from occurring unchecked and unethically. Publicizing this practice will also allow a broader discussion of its ethical ramifications, impacts, and effects on medical training. This will let us determine the true need for this practice and the best methods of teaching procedural skills, especially as technology continues to improve. Given the public’s general willingness and acceptance of this practice, publicizing it to general society is also imperative. In turn, this will protect their trust in the medical profession and ensure that the practice continues to occur ethically with consideration towards the patient’s wishes. Once publicized, an “opt-in” consent process equivocal to that of organ donation or anatomical donation should be enacted for this practice, with the provision of documentation indicating the donor’s status. In this manner, fully informed consent can be obtained well before the practice occurs and further publicize its existence, preventing emotional turmoil for patients and their family members. Families should also be reimbursed after all procedures and studies are completed (similar to that of anatomical donation), given that their loved ones altruistically helped to further medical education and training.

While many may consider banning this practice, the consequences of doing so must be considered. Preventing trainees from practicing life-sustaining procedures while fully adequate models do not exist may lead to the development of poorly trained physicians who are not able to follow the tenets of “do no harm.” Making this practice illegal may lead to further secrecy and ethical violations under a lack of oversight, creating further complications from unsuccessful procedures. Instead, permitting this practice would allow for discussion regarding informed consent, respect for the deceased, and its ethicality. This would parallel the use of the Pernkopf Atlas in anatomy, where the unethical use of anatomical drawings of victims of the Nazi regime can be used as an ethical teaching point.

## Conclusions

Today, physicians constantly face a juxtaposition between non-maleficence and beneficence as they fulfill their obligations towards relieving suffering, treating maladies, and improving their patients' quality of life. The desire to perfect their ability to perform their obligations further serves as a cornerstone in allowing trainees to perform postmortem procedures that do not benefit the patient. Allowing healthcare professionals to perform posthumous procedures on the newly deceased will ultimately decrease patients’ morbidity and mortality as they gain and perfect their procedural skills. 

While the number of postmortem procedures performed is severely under-reported, the continuance and advocation of this practice have significant ethical ramifications that trainees must consider while striving to perfect their procedural skills. Without a proper consent process and educational setting for this practice, the damage to the doctor-patient relationship can erode trainees’ empathy, allow a disrespectful attitude towards human life to develop, and create significant mistrust for families with the medical system. A formal consent process based on existing frameworks will allow all concerns to be addressed while obtaining a proper, holistic understanding of the practice from a training and ethical perspective. Publicizing this practice will also allow a broader discussion of its effects and impacts on medical training while preserving trust in the medical profession. Legitimizing posthumous procedures in this manner will thus give trainees one more way to save lives without creating significant harm.
